# Association of Depression and Anxiety with Cardiac Structural and Functional Characteristics in Heart Failure with Reduced and Mildly Reduced Ejection Fraction

**DOI:** 10.3390/clinpract13020036

**Published:** 2023-03-09

**Authors:** Timea Magdolna Szabo, Előd Ernő Nagy, Ádám Kirchmaier, Erhard Heidenhoffer, Hunor-László Gábor-Kelemen, Marius Frăsineanu, Attila Frigy

**Affiliations:** 1 Department of Biochemistry and Environmental Chemistry, George Emil Palade University of Medicine, Pharmacy, Science and Technology of Târgu Mureș, 540142 Târgu Mureș, Romania; 2 Laboratory of Medical Analysis, Clinical County Hospital Mureș, 540394 Târgu Mureș, Romania; 3 Department of Cardiology, Clinical County Hospital Mureș, 540103 Târgu Mureș, Romania; 4 Department of Internal Medicine IV, George Emil Palade University of Medicine, Pharmacy, Science and Technology of Târgu Mureș, 540103 Târgu Mureș, Romania

**Keywords:** depression, anxiety, heart failure, heart failure with reduced ejection fraction, heart failure with mildly reduced ejection fraction

## Abstract

Heart failure and mental health conditions frequently coexist and have a negative impact on health-related quality of life and prognosis. We aimed to evaluate depression and anxiety symptoms and to determine the association between psychological distress and cardiac parameters in heart failure with reduced and mildly reduced ejection fraction. A total of 43 patients (33 male, mean age 64 ± 10 years) with heart failure and left ventricular systolic dysfunction (29 with HFrEF, 14 with HFmrEF) underwent comprehensive echocardiographic evaluation. All study participants completed questionnaires for the assessment of depression (PHQ-9), anxiety (GAD-7), and health-related quality of life (MLHFQ). Ten (34%) patients with HFrEF and two (14%) participants with HFmrEF had moderate-to-severe depression symptoms. Significant anxiety symptoms were more frequent in HFrEF (10 vs. 2 patients; 34% vs. 14%). Poor quality of life was also more common among patients with HFrEF (17 vs. 5 patients; 59% vs. 36%), showing higher MLHFQ scores (*p* = 0.009). Moreover, PHQ-9, GAD-7, and MLHFQ scores showed significant correlations between NYHA class severity and the presence of peripheral edema. The symptoms of dyspnea correlated with both PHQ-9 and MLHFQ scores. Significant correlations were observed between MLHFQ scores and a large number of clinical features, such as exercise capacity, 6MWT distance, the need for furosemide, echocardiographic parameters (LVEDVI, LVESVI, LVEF, LVGLS, SVI), and laboratory variables (albumin, GFR, NT-proBNP). In the multiple linear regression analysis, dyspnea proved to be a significant predictor of higher PHQ-9 and MLHFQ scores, even after adjusting for potential confounders. High symptom burden due to psychological distress is common among patients with HFrEF and HFmrEF. More efficient control of congestion may improve depression, anxiety symptoms, and health-related quality of life.

## 1. Introduction

Heart failure (HF) remains a global healthcare challenge, with multimorbidity and the associated treatment burden affecting most patients. Although currently available guideline-directed medical and device therapy significantly improved morbidity and mortality, poor prognosis and low quality of life (QOL) remain further challenges [1,2].

Depression affects up to 20% of HF patients, negatively influencing disease outcomes and emotional well-being [1]. Both depression and HF show similar pathophysiological pathways, such as inflammation, increased platelet reactivity, arrhythmias, neuroendocrine dysregulation, high-risk behaviors, including smoking, poor physical health, and low adherence to treatment [3]. Depression has a negative impact on QOL, regardless of functional status, and emerged as an independent risk factor of poor outcomes in HF [3]. Current ESC HF guidelines provide recommendations for depression screening using validated questionnaires [1]. Medication, exercise training, and cognitive-behavioral therapy (CBT) improve depression symptoms in HF patients [3]. Moreover, left ventricular (LV) systolic dysfunction is predictive of depression and symptom severity in patients with myocardial infarction [4,5].

Other mental health conditions, such as anxiety, should also be promptly recognized and treated in HF [1]. Symptoms of anxiety are more common in HF with preserved ejection fraction (HFpEF) than in HF with reduced ejection fraction (HFrEF) [6]. Both anxiety and depression are reliable predictors of QOL in HFrEF [7].

Anxiety and depression worsen not only HF but also cardiovascular disease generally. In fact, anxiety proved to be a better predictor of serotonin-mediated platelet reactivity than depression in patients with stable coronary artery disease on dual antiplatelet therapy with clopidogrel plus aspirin three months after an acute coronary syndrome [8].

Adverse LV remodeling has a negative impact on morbidity and mortality in patients with HF [9]. LV volumes (especially LVESV) predict HF outcomes even after adjusting for LVEF and infarct size [10]. Moreover, the short-term benefits of drug or device therapy on LV remodeling, as assessed by changes in LVEF, LVESV, and LVEDV, are associated with better longer-term outcomes [11]. Sodium-glucose cotransporter 2 inhibitors improve HF beyond their glucoretic and natriuretic effect, by enhancing myocardial energetics [12,13]. Empaglifozin ameliorates LV remodeling, improves functional capacity [14], reduces mortality and hospitalization [15], and increases QOL [14,16].

The aim of our study was to evaluate symptom burden due to psychological distress in HFrEF and HF with mildly reduced ejection fraction (HFmrEF), and to determine the relationship between depression and anxiety and heart structure and function as assessed by echocardiography, and other clinical and laboratory parameters. Moreover, we tried to identify cardiac ultrasound parameters indicative of depression and anxiety in HFrEF and HFmrEF, in order to find subgroups of patients at higher risk of developing mental health conditions for targeted screening.

## 2. Materials and Methods

### 2.1. Study Cohort

Forty-three patients with left ventricular ejection fraction (LVEF) < 50% were enrolled in a prospective, single-center study, during January and November 2022 in Târgu Mureș, Romania. Inclusion criteria were the diagnosis of HFrEF or HFmrEF according to currently available guidelines [1], New York Heart Association (NYHA) class I-III, hemodynamic stability (regarding heart rate, blood pressure, clinical congestion), and willingness to participate. Exclusion criteria were signs and symptoms of infection, poor kidney function (estimated glomerular filtration rate (GFR) < 20 mL/min/1.73 m^2^ using the Chronic Kidney Disease Epidemiology Collaboration (CKD-EPI) equation), known liver disease, autoimmune disorders, and cancer. The recruited patients were either outpatients or inpatients hospitalized for worsening heart failure, assessed after decongestion and right before discharge. The outpatients were on standard HF medication for the last 3 months. Each participant underwent systematic data collection regarding routine demographic, clinical, laboratory, and echocardiographic data. A lung ultrasound (LUS) was also performed for the assessment of pulmonary congestion. Patients were screened for depression symptoms and anxiety. Health-related quality of life (HRQL) was also evaluated.

The study complies with the Declaration of Helsinki. The institutional ethics committee approved the study protocol (7716/02.07.2021, Clinical County Hospital Mureș), and participants provided written informed consent before recruiting.

### 2.2. Cardiac and Lung Ultrasound

Echocardiography was carried out using a Philips Epiq7 ultrasound machine (Philips Ultrasound, Inc., Bothell, WA, USA) and a Philips X5-1 xMATRIX array transducer (1–5 MHz) according to current recommendations [17,18,19]. LVEF was assessed using the biplane method of disks (modified Simpson’s method). Global longitudinal strain measurements were made in the three standard apical views and then averaged. LV and left atrial (LA) volumes were acquired by 2D echocardiography and normalized by body surface area (BSA). Averaged e’ from medial and lateral sites of the mitral annulus was used for the calculation of E/e’.

An eight-zone LUS protocol was performed on every participant. Each hemithorax was divided into four zones assessing the anterior and lateral chest and scanning for B-lines [20]. Patients were evaluated in a semirecumbent position, using the same cardiac probe (Philips X5-1 xMATRIX, 1–5 MHz) positioned perpendicular to the ribs. Imaging depth was tailored to the patient. All quadrants were screened, and the following threshold was applied: normal (0–2 B-lines/zone) and abnormal (≥3 B-lines/zone) [20].

### 2.3. Six-Minute Walking Test and Ankle-Brachial Index Assessment

For further risk stratification, the six-minute walking (6MWT) distance was also measured based on available guidelines [21].

Ankle-brachial index testing using standard peripheral CW Doppler (5 MHz) was carried out for the assessment of subclinical peripheral arterial disease (PAD), and a threshold of ≤0.9 was used.

### 2.4. Mental Health Status and Quality of Life in Heart Failure

The Patient Health Questionnaire-9 (PHQ-9) and Generalized Anxiety Disorder-7 (GAD-7) were applied to evaluate coexisting depression and anxiety [22,23,24]. Depression symptoms were categorized using PHQ-9 scores, and values ≥ 10 were considered significant [23]. The same cut-off values were established for anxiety symptoms as determined by GAD-7 (scores ≥ 10 were considered relevant) [24]. HRQL was assessed with Minnesota Living with Heart Failure Questionnaire (MLHFQ), comprising 21 items, which addressed emotional, physical, and socioeconomic aspects of heart failure [25]. Scores ≥ 24 on the MLHFQ were considered consistent with a moderate-to-poor HRQL [26].

### 2.5. Laboratory Data

Blood samples were collected after 12-h fasting in red-top and purple-top tubes (Becton-Dickinson Vacutainer Systems, Wokingham, Berkshire, UK). The tubes without additives were centrifuged at 3000 rpm for 10 min to separate the serum. Total-, LDL-, and HDL-cholesterol, serum triglycerides, creatinine, albumin, serum iron, ferritin, and C-reactive protein (CRP) levels were measured with commercial biochemical kits on an Arhitect C4000 (Abbott Laboratories, Diagnostic Division, Abbott Park, IL, USA). Plasma fibrinogen was determined using a coagulometric method with Multifibren U reagent on Sysmex CA-1500 (Sysmex Corporation, Kobe, Japan). Serum N-terminal pro B-type natriuretic peptide levels (NT-proBNP) were measured using an electrochemiluminescent immunoassay on Elecsys 2010 (Roche Diagnostics International, Rotkreuz, Switzerland). Tripotassium ethylenediaminetetraacetic acid (K3 EDTA) precoated tubes were used for complete blood count analysis on a Mindray BC6000 (Mindray Global, Shenzhen, China).

### 2.6. Statistical Analysis

Data distribution was assessed using Kolmogorov–Smirnov and Shapiro–Wilk tests. Paired t-test was performed for variables showing Gaussian distribution. The Mann–Whitney U test and Spearman rank correlation analysis were applied for variables showing skewed distribution. Categorical variables were quantified for absolute and relative frequency and the 2 × 2 or 3 × 2 contingency tables were analyzed with the χ^2^ test. *p*-values < 0.05 were considered statistically significant. Data processing was performed using Microsoft Excel 2016 (Microsoft Corporation, Redmond, WA, USA) and GraphPad Prism 9.5.0 (GraphPad Software LLC., San Diego, CA, USA).

## 3. Results

### 3.1. Study Group Characteristics

Twenty-nine patients with HFrEF and fourteen patients with HFmrEF were included in the study (Table 1 and Table 2). A significantly higher number of participants with HFrEF were recruited before discharge, rather than from ambulatory care (22 (69%) vs. 2 (14%), *p* = 0.004). Patients with HFmrEF showed better exercise capacity (*p* = 0.049), had higher levels of serum iron (*p* = 0.015), lower NT-proBNP values (*p* = 0.015), and a less frequent need for furosemide for symptom relief (*p* = 0.036). Study participants with HFrEF had increased LA volume indexes (LAVI), LV end-diastolic and end-systolic volume indexes (LVEDVI, LVESVI), and low stroke volume indexes (SVI).

**Figure 1:**
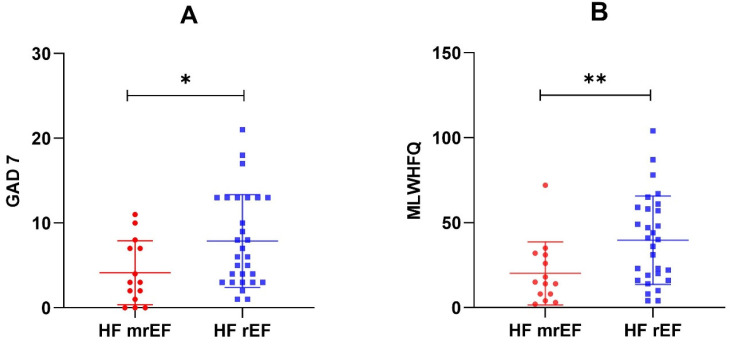
GAD-7 scores (**A**) and MLHFQ scores (**B**) in the two EF subgroups. * *p* = 0.026; ** *p* = 0.009.

Ten (34%) patients with HFrEF had moderate-to-severe depression symptoms, compared to only two (14%) participants with HFmrEF. Significant anxiety symptoms were also more frequent in HFrEF: 10 (34%) vs. 2 (14%). Reduced HRQL was more common among patients with HFrEF: 17 (59%) vs. 5 (36%), showing higher MLHFQ scores (*p* = 0.009) (Figure 1).

### 3.2. Correlations for GAD-7, PHQ-9, and MLHFQ

Table 3 summarizes the association between questionnaire scores and several parameters. GAD-7, PHQ-9, and MLHFQ scores showed significant correlations with NYHA class severity and the presence of peripheral edema. Symptoms of dyspnea correlated with both PHQ-9 and MLHFQ scores. PHQ-9 scores also showed significant correlations with echocardiographic parameters, such as LAVI and SVI. Significant correlations were observed between HRQL scores and a large number of clinical and paraclinical characteristics: exercise capacity, 6MWT distance, the need for loop diuretics, cardiac ultrasound imaging measurements–LVEDVI, LVESVI, LVEF, LVGLS, SVI, and different laboratory variables–albumin, GFR, NT-proBNP.

GAD-7 scores showed statistically significant positive correlations with PHQ-9 scores (Spearman’s R = 0.608, *p* < 0.0001) and MLHFQ scores (Spearman’s R = 0.521, *p* = 0.0003). PHQ-9 scores also correlated with MLHFQ scores (Spearman’s R = 0.683, *p* < 0.0001).

### 3.3. Multivariate Linear Regression Models for PHQ-9 and MLHFQ

Multiple linear regression models were set up for the determinants of PHQ-9 scores as a continuous variable. In the first model, the symptom of dyspnea proved to be the only significant predictor, which remained significant, even when adjusted for the presence of peripheral edema, LAVI, LVGLS, and SVI (Model 1, Table 4a). Two different models were constructed for MLHFQ (Table 4b). The presence of dyspnea also predicted MLHFQ scores, maintaining its significance when adjusted for LAVI, LVEDVI, LVESVI, LVEF (Model 2), and peripheral edema, LVESVI, LVEF, exercise capacity, and serum albumin (Model 3).

## 4. Discussion

Depression is common among patients with HF, negatively affecting healthcare-related economic burdens [3]. Two meta-analyses conducted 10 years apart showed an increasing prevalence of depression in HF—21.5% vs. 29% [27,28]. Age might also play an important role in the frequency of depression symptoms and their impact on mortality. One-year mortality rates were significantly higher in elderly patients (≥70 years), who were associated with higher Beck Depression Inventory scores [29]. We found no significant correlations between age and PHQ-9 scores, but our study population was younger with a mean age of 64 ± 10 years.

The relationship between HF and depression is bidirectional—depression increases the risk of developing HF, especially in specific populations, whereas patients with HF frequently associate comorbid depression, both shown to negatively impact HRQL. Moreover, HF and depression share similar pathophysiological mechanisms, and the inflammatory pathway is considered one of great importance [3]. We failed to find significant correlations between PHQ-9 scores and biomarkers of inflammation, such as neutrophil-to-lymphocyte ratio, CRP, and fibrinogen. Neutrophil-to-lymphocyte ratios of 1.57 or higher proved to be predictors of severe depression in patients diagnosed with depression [30]. Our patients had median neutrophil-to-lymphocyte ratios of 2.3 (1.89–3.08), but values showed no association with PHQ-9 scores.

Anxiety and depression symptoms are significantly more common in outpatients with HFpEF when compared to HFrEF [6]. Significant anxiety symptoms using the State-Trait Anxiety Inventory were reported in HFrEF (31.6% and 27.2%) [31]. Moreover, 34% of our patients with HFrEF had moderate-to-severe anxiety symptoms. Trait anxiety failed to show prognostic value in predicting all-cause mortality at 18 months, all-cause death, or readmission, and cardiovascular mortality or readmission in HFrEF, but not in HFpEF [31].

NYHA class showed significant positive correlations with all assessed scores (GAD-7, PHQ-9, MLHFQ). Dyspnea remained significant for predicting depression symptoms and QOL even after adapting for confounders. Moreover, MLHFQ scores correlated with the need for furosemide and NT-proBNP levels. Residual congestion estimated by LUS in decompensated HF was associated with increased all-cause mortality during a 4-year follow-up [32]. This highlights the importance of patient management after discharge and during regular follow-ups, in order to achieve decongestion and prevent episodes of cardiac decompensation and disease progression.

We established LAVI and SVI as potential predictors for depression symptoms. These echocardiographic parameters might help healthcare professionals to identify patients at higher risk of developing depression in HFrEF and HFmrEF. In the MIND-IT study, patients with myocardial infarction and lower EF presented a higher risk of developing depression, especially men [4]. In our study cohort, LVEF (*p* = 0.093) and LVGLS (*p* = 0.067) showed a tendency towards a stronger increase in depression symptom scores but did not reach statistical significance.

Depression and 6MWT distance were associated with poor quality of life in patients with advanced HF, who were candidates for left ventricular assist devices [33]. We found significant correlations between exercise capacity, 6MWT distance, and MLHFQ scores. The HF-ACTION trial randomized patients with HF and NYHA class I-IV, LVEF ≤ 35%, to aerobic exercise training or education and guideline-directed therapy, with exercise leading to a significant reduction in depression symptoms at 3 and 12 months [34]. In a meta-analysis on CBT in HF and comorbid depression, patients reported improvement in depression symptoms and QOL after finishing treatment sessions [35].

The routine prescription of pharmacotherapy for depression in HF should be withheld. In the double-blind, placebo-controlled randomized MOOD-HF clinical trial, which included participants with HF and NYHA class II-IV, LVEF < 45%, treatment with escitalopram for 18 months showed no significant reduction in all-cause mortality or hospitalization, nor improvement in depression symptoms [36]. Currently, available ESC guidelines for HF management recommend avoiding the treatment of depression with tricyclic antidepressants because of their possible side effects [1].

## 5. Conclusions

Depression and anxiety symptoms are prevalent among patients with HFrEF and HFmrEF. GAD-7 and MLHFQ scores are significantly higher in HFrEF, indicating greater symptom burden. Dyspnea proved to be a significant predictor of depression symptoms and HRQL in HF with left ventricular systolic dysfunction. A number of echocardiographic parameters showed significant correlations with PHQ-9 and MLHFQ scores, thus identifying a subgroup of patients more vulnerable to developing depression for future targeted screening.

## Figures and Tables

**Table 1:** Clinical, laboratory, and ultrasound variables in HF patients.

Characteristics	HF with Impaired EF, n = 43	HFrEF, n = 29	HFmrEF, n = 14	*p*-Value
**Basic characteristics, comorbidities**				
Inpatient/outpatient, no. (%)	22 (51)/21 (49)	20 (69)/9 (31)	2 (14)/12 (84)	0.004
Male/female, no. (%)	33 (76)/10 (24)	22 (76)/7 (24)	11 (78)/3 (22)	0.895
Age, mean ± SD (min; max), years	64 ± 10 (40; 86)	64 ± 10 (40; 86)	64 ± 10 (43; 78)	0.859
BMI, mean ± SD, kg/m^2^	28.51 ± 5.55	29.26 ± 5.76	26.95 ± 4.92	0.205
Heart disease, median (IQR), years	8 (5–15)	9 (6–16)	8 (3–11)	0.174
HF, median (IQR), years	3 (1–8)	4 (1–8)	3 (1–8)	0.846
NYHA class I/II/III, no. (%)	4 (9)/28 (65)/11 (26)	1 (3)/19 (66)/9 (31)	3 (22)/9 (64)/2 (14)	0.131
Smoking, no. (%)	13 (30)	10 (34)	3 (22)	0.493
Hypertension, no. (%)	24 (56)	15 (51)	9 (64)	0.512
Cardiomyopathy ischemic/non-ischemic, no. (%)	15 (35)/28 (65)	13 (45)/16 (55)	2 (14)/12 (86)	0.105
Mitral valve regurgitation medium/severe, no. (%)	11 (26)	9 (31)	2 (14)	0.375
Aortic stenosis medium/severe, no. (%)	3 (7)	1 (3)	2 (14)	0.566
Tricuspid regurgitation medium/severe, no. (%)	10 (24)	7 (24)	3 (22)	0.895
AF/AFL, no. (%)	16 (37)	11 (38)	5 (36)	0.916
PVCs/NSVT, no. (%)	20 (47)	13 (45)	7 (50)	0.793
ICD/CRT, no. (%)	2 (5)	1 (3)	1 (3)	0.851
LBBB, no. (%)	21 (49)	16 (55)	5 (36)	0.307
Type II DM, no. (%)	8 (19)	7 (24)	1 (3)	0.365
Atherosclerosis 0/1/2/3 territories *, no. (%)	23 (53)/9 (21)/11 (26)/0 (0)	12 (41)/6 (21)/11 (38)/0 (0)	11 (78)/3 (22)/0 (0)/0 (0)	0.017
Symptomatic PAD, no. (%)	5 (12)	4 (14)	1 (3)	0.728
ABI, mean ± SD	1.05 ± 0.24	1.03 ± 0.25	1.09 ± 0.19	0.492
CAD, no. (%)	8 (19)	7 (24)	1 (7)	0.365
COPD, no. (%)	9 (21)	6 (21)	3 (22)	0.979
Iron deficiency, no. (%)	11 (26)	8 (28)	3 (22)	0.751
Anemia, no. (%)	4 (9)	3 (10)	1 (7)	0.872
Thyroid function 0/1/2 **, no. (%)	38 (89)/4 (9)/1 (2)	26 (90)/2 (7)/1 (3)	12 (86)/2 (14)/0 (0)	0.862
**Ultrasound parameters**				
Lung US profile 0/1, no. (%)	32 (74)/11 (26)	19 (66)/10 (34)	13 (93)/1 (7)	0.144
LAVI, median (IQR), mL/m^2^	42.51 (33.23–50.64)	45.4 (39.4–55.09)	31.99 (28.31–37.95)	0.0008
E/e’, median (IQR)	9.8 (7.97–12.4)	10.15 (8.4–12.8)	8.85 (6.86–11.65)	0.139
LVEDVI, mean ± SD, mL/m^2^	94 ± 34	106 ± 32	67 ± 16	<0.0001
LVESVI, mean ± SD, mL/m^2^	62 ± 30	74 ± 28	36 ± 10	<0.0001
LVEF, mean ± SD, %	35 ± 9	29 ± 6	46 ± 3	<0.0001
LVGLS, mean ± SD, %	−10.58 ± 3.52	−8.81 ± 2.63	−14.25 ± 1.91	<0.0001
SVI, median (IQR), mL/m^2^	31.57 (25.23–40.1)	27.55 (24.15–31.7)	38.35 (35.47–45.51)	0.004
**Laboratory variables**				
WBC, mean ± SD, ×1000/mL	7.43 ± 1.89	7.43 ± 2.03	7.45 ± 1.62	0.973
Neu/lym, median (IQR)	2.3 (1.89–3.08)	2.47 (1.96–3.09)	2.2 (2.08–2.87)	0.496
Hemoglobin, mean ± SD, g/dL	14.9 ± 1.83	15.03 ± 2.08	14.62 ± 1.18	0.499
Ferritin, mean ± SD, ng/dL	185.91 ± 180.26	201.12 ± 208.85	154.41 ± 97.17	0.948
Iron, mean ± SD, μg/dL	85.95 ± 31.63	77.48 ± 28.44	103.5 ± 31.62	0.015
Cholesterol, mean ± SD, mg/dL	174.13 ± 47.59	175.1 ± 51.17	172.14 ± 40.88	0.851
HDL-cholesterol, mean ± SD, mg/dL	43.5 ± 12.6	41.98 ± 12.28	46.65 ± 13.13	0.260
LDL-cholesterol, mean ± SD, mg/dL	104.39 ± 35.68	105.08 ± 37.16	102.95 ± 33.69	0.857
Triglycerides, median (IQR), mg/dL	112 (81.5–139)	112 (83–141)	105 (79–136)	0.543
CRP, median (IQR), mg/Dl	0.34 (0.13–0.71)	0.35 (0.2–0.75)	0.25 (0.08–0.38)	0.233
Fibrinogen, mean ± SD, mg/dL	394.41 ± 134.16	413.55 ± 134.41	354.76 ± 129.35	0.181
Albumin, median (IQR), g/L	44.6 (41.65–46.95)	44.2 (41.6–46)	45.65 (43.25–47.85)	0.182
GFR, mean ± SD, mL/min/m^2^	75 ± 18	71 ± 17	82 ± 17	0.082
NT-proBNP, median (IQR), pg/mL	992.7 (524.4–1733.45)	1113 (686.6–2664.9)	602.55 (251.07–1211.37)	0.015
**Treatment**				
Loop diuretic, no. (%)	33 (76)	26 (90)	7 (50)	0.036
MRA, no. (%)	38 (89)	25 (86)	13 (93)	0.728
SGLT2i, no. (%)	26 (60)	19 (66)	7 (50)	0.416
BB, no. (%)	39 (91)	27 (93)	12 (86)	0.699
ACEI/ARB, no. (%)	8 (19)	5 (17)	3 (22)	0.832
ARNI, no. (%)	29 (67)	19 (66)	10 (71)	0.762
Statin and/or ezetimibe, no. (%)	27 (63)	19 (66)	8 (57)	0.665

BMI, body mass index; HF, heart failure; NYHA, New York Heart Association; AF, atrial fibrillation; AFL, atrial flutter; PVCs, premature ventricular contractions; NSVT, non-sustained ventricular tachycardia; ICD, implantable cardioverter defibrillator; CRT, cardiac resynchronization therapy; LBBB, left bundle branch block; DM, diabetes mellitus; PAD, peripheral artery disease; ABI, ankle-brachial index; CAD, carotid artery disease; COPD, chronic obstructive pulmonary disease; US, ultrasound; LAVI, left atrial volume index; LVEDVI, left ventricular end-diastolic volume index; LVESVI, left ventricular end-systolic volume index; LVEF, left ventricular ejection fraction; LVGLS, left ventricular global longitudinal strain; SVI, stroke volume index; WBC, white blood cells; Neu/lym, neutrophil-to-lymphocyte ratio; CRP, C-reactive protein; GFR, glomerular filtration rate; NT-proBNP, N-terminal pro B-type natriuretic peptide; MRA, mineralocorticoid receptor antagonist; SGLT2i, sodium-glucose cotransporter-2 inhibitor; BB, beta-blocker; ACEI, angiotensin-converting enzyme inhibitor; ARB, angiotensin II receptor blocker; ARNI, angiotensin receptor/neprilysin inhibitor. * Coronary artery disease, symptomatic and asymptomatic peripheral artery disease, carotid artery disease; ** 0—normal thyroid function, 1—hypothyroidism, 2—hyperthyroidism.

**Table 2:** Mental Health and HRQL in HF patients.

Characteristics	HF with Impaired EF, n = 43	HFrEF, n = 29	HFmrEF, n = 14	*p*-Value
GAD-7 score, mean ± SD	7 ± 5	8 ± 5	4 ± 4	0.026
PHQ-9 score, mean ± SD	8 ± 5	9 ± 6	6 ± 4	0.061
Problem severity 0/1/2/3 *, no. (%)	10 (24)/31 (72)/1 (2)/1 (2)	6 (21)/21 (73)/1 (3)/1 (3)	4 (29)/10 (71)/0 (0)/0 (0)	0.501
MLHFQ score, mean ± SD	33 ± 25	40 ± 26	20 ± 19	0.009
Dyspnea, no. (%)	38 (89)	27 (93)	11 (78)	0.441
Palpitations, no. (%)	11 (26)	7 (24)	4 (29)	0.823
Peripheral edema, no. (%)	12 (28)	11 (38)	1 (7)	0.100
Exercise 1/2/3 **, no. (%)	6 (14)/10 (23)/27 (63)	6 (21)/8 (28)/15 (51)	0 (0)/2 (14)/12 (86)	0.049
6MWT, median (IQR), m	405 (252–488)	405 (250–452)	445.5 (362.75–523.75)	0.103

GAD-7, Generalized Anxiety Disorder-7; PHQ-9, Patient Health Questionnaire-9; MLHFQ, Minnesota Living with Heart Failure Questionnaire; 6MWT, 6-min walking test distance. * As assessed by PHQ-9 last item “If you checked off any problems, how difficult have these problems made it for you to do your work, take care of things at home, or get along with other people?”, 0—not difficult at all, 1—somewhat difficult, 2—very difficult, 3—extremely difficult; ** 1—<30 min exercise/week, 2—30–120 min exercise/week, 3—>120 min exercise/week.

**Table 3:** Correlations for GAD-7, PHQ-9, and MLHFQ scores in the overall group (n = 43).

Characteristics	GAD-7 Score		PHQ-9 Score		MLHFQ Score	
	Spearman’s R	*p*-Value	Spearman’s R	*p*-Value	Spearman’s R	*p*-Value
Age	0.083	0.594	−0.144	0.356	−0.087	0.576
NYHA class	0.306	0.045	0.411	0.006	0.444	0.002
Peripheral edema	0.310	0.042	0.302	0.048	0.405	0.006
Dyspnea	0.185	0.234	0.504	0.0005	0.435	0.003
Exercise	−0.140	0.367	−0.139	0.370	−0.333	0.029
6MWT	−0.253	0.367	−0.302	0.370	−0.287	0.029
Loop diuretics	0.051	0.744	0.289	0.059	0.355	0.019
Lung US profile	0.131	0.400	0.194	0.212	0.244	0.113
LVEDVI	0.102	0.515	0.121	0.438	0.381	0.011
LVESVI	0.121	0.436	0.137	0.377	0.400	0.007
LVEF	−0.215	0.166	−0.259	0.093	−0.467	0.001
LVGLS	0.201	0.194	0.281	0.067	0.487	0.0009
SVI	−0.308	0.430	−0.321	0.043	−0.292	0.041
LAVI	−0.253	0.100	−0.302	0.048	−0.287	0.061
E/e’	0.111	0.478	−0.001	0.992	0.164	0.290
Neu/lym	−0.010	0.944	0.028	0.857	−0.002	0.987
CRP	−0.050	0.748	0.210	0.175	0.127	0.414
Fibrinogen	−0.003	0.983	0.066	0.671	0.093	0.552
Hemoglobin	−0.089	0.566	0.025	0.869	0.210	0.174
Ferritin	−0.109	0.483	−0.065	0.675	−0.251	0.103
Iron	−0.143	0.356	−0.117	0.452	−0.289	0.059
Albumin	−0.0006	0.996	−0.171	0.272	−0.320	0.036
GFR	−0.223	0.150	−0.166	0.286	−0.425	0.004
NT-proBNP	0.229	0.138	0.263	0.087	0.418	0.005
Cholesterol	0.286	0.062	0.129	0.407	−0.004	0.975
HDL-cholesterol	0.102	0.512	−0.005	0.970	−0.027	0.862
LDL-cholesterol	0.201	0.195	0.015	0.920	−0.140	0.370
Triglycerides	0.113	0.469	0.153	0.324	0.139	0.371

NYHA, New York Heart Association; 6MWT, 6-min walking test distance; US, ultrasound; LVEDVI, left ventricular end-diastolic volume index; LVESVI, left ventricular end-systolic volume index; LVEF, left ventricular ejection fraction; LVGLS, left ventricular global longitudinal strain; SVI, stroke volume index; LAVI, left atrial volume index; Neu/lym, neutrophil-to-lymphocyte ratio; CRP, C-reactive protein; GFR, glomerular filtration rate; NT-proBNP, N-terminal pro B-type natriuretic peptide.

**Table 4:** **(a).** Multivariate linear regression analysis of the factors correlated with PHQ-9 scores in the overall patient group. **(b)**. Multivariate linear regression analysis of the factors correlated with MLHFQ scores in the overall patient group (n = 43).

**(a)**					
**Model 1. Summary of regression**					
**Variables**	**B**	**SD of B**	**β**	**t**	***p*-Value**
Dyspnea	5.633	2.446	0.345	2.303	0.027
Peripheral edema	2.295	1.930	0.197	1.189	0.242
LAVI	−0.008	0.006	−0.192	−1.240	0.223
LVGLS	0.122	0.281	0.081	0.432	0.668
SVI	0.012	0.076	0.024	0.157	0.876
**(b)**					
**Model 2. Summary of regression**					
**Variables**	**B**	**SD of B**	**β**	**t**	***p*-Value**
Dyspnea	24.042	11.339	0.308	2.120	0.041
LAVI	−0.053	0.032	−0.273	−1.664	0.104
LVEDVI	0.590	0.523	0.781	1.128	0.267
LVESVI	−0.549	0.706	−0.652	−0.778	0.442
LVEF	−0.624	0.897	−0.225	−0.695	0.491
**Model 3. Summary of regression**					
**Variables**	**B**	**SD of B**	**β**	**t**	***p*-Value**
Dyspnea	23.290	11.335	0.298	2.055	0.047
Peripheral edema	14.346	9.377	0.257	1.530	0.135
Exercise	−9.366	5.354	−0.272	−1.749	0.089
LVESVI	0.192	0.201	0.227	0.953	0.347
LVEF	0.221	0.810	0.080	0.273	0.787
Albumin	−0.147	0.984	−0.023	−0.149	0.882

LAVI, left atrial volume index; LVGLS, left ventricular global longitudinal strain; SVI, stroke volume index; LVEDVI, left ventricular end-diastolic volume index; LVESVI, left ventricular end-systolic volume index; LVEF, left ventricular ejection fraction.

## Data Availability

Publicly archived datasets analyzed during the study can be found under the following link: https://doi.org/10.6084/m9.figshare.21895845.v1, accessd on 13 January 2023.

## Limitations

The main limitation of our study is the small number of participants. Also, baseline differences between the two groups may influence the results. Therefore, larger studies are required to provide more accurate and reliable results.

## References

[1] McDonagh T.A., Metra M., Adamo M., Gardner R.S., Baumbach A., Bohm M., Burri H., Butler J., Celutkiene J., Chioncel O. (2021). 2021 ESC Guidelines for the diagnosis and treatment of acute and chronic heart failure. Eur. Heart J..

[2] Heidenreich P.A., Bozkurt B., Aguilar D., Allen L.A., Byun J.J., Colvin M.M., Deswal A., Drazner M.H., Dunlay S.M., Evers L.R. (2022). 2022 AHA/ACC/HFSA Guideline for the Management of Heart Failure: Executive Summary: A Report of the American College of Cardiology/American Heart Association Joint Committee on Clinical Practice Guidelines. Circulation.

[3] Sbolli M., Fiuzat M., Cani D., O′Connor C.M. (2020). Depression and heart failure: The lonely comorbidity. Eur. J. Heart Fail..

[4] van Melle J.P., de Jonge P., Ormel J., Crijns H., van Veldhuisen D.J., Honig A., Schene A.H., van den Berg M.P., Investigators M.-I. (2005). Relationship between left ventricular dysfunction and depression following myocardial infarction: Data from the MIND-IT. Eur. Heart J..

[5] Bagherian-Sararoudi R., Gilani B., Bahrami Ehsan H., Sanei H. (2013). Relationship between left ventricular ejection fraction and depression following myocardial infarction: An original article. ARYA Atheroscler..

[6] Bekfani T., Nisser J., Derlien S., Hamadanchi A., Frob E., Dannberg G., Lichtenauer M., Smolenski U.C., Lehmann G., Mobius-Winkler S. (2021). Psychosocial factors, mental health, and coordination capacity in patients with heart failure with preserved ejection fraction compared with heart failure with reduced ejection fraction. Esc. Heart Fail..

[7] Fino P., Sousa R.M., Carvalho R., Sousa N., Almeida F., Pereira V.H. (2020). Cognitive performance is associated with worse prognosis in patients with heart failure with reduced ejection fraction. Esc. Heart Fail..

[8] Zafar M.U., Paz-Yepes M., Shimbo D., Vilahur G., Burg M.M., Chaplin W., Fuster V., Davidson K.W., Badimon J.J. (2010). Anxiety is a better predictor of platelet reactivity in coronary artery disease patients than depression. Eur. Heart J..

[9] Konstam M.A., Kramer D.G., Patel A.R., Maron M.S., Udelson J.E. (2011). Left Ventricular Remodeling in Heart Failure Current Concepts in Clinical Significance and Assessment. JACC-Cardiovasc. Imaging.

[10] White H.D., Norris R.M., Brown M.A., Brandt P.W.T., Whitlock R.M.L., Wild C.J. (1987). Left-Ventricular End-Systolic Volume as the Major Determinant of Survival after Recovery from Myocardial-Infarction. Circulation.

[11] Kramer D.G., Trikalinos T.A., Kent D.M., Antonopoulos G.V., Konstam M.A., Udelson J.E. (2010). Quantitative Evaluation of Drug or Device Effects on Ventricular Remodeling as Predictors of Therapeutic Effects on Mortality in Patients With Heart Failure and Reduced Ejection Fraction A Meta-Analytic Approach. J. Am. Coll. Cardiol..

[12] Selvaraj S., Fu Z.X., Jones P., Kwee L.C., Windsor S.L., Ilkayeva O., Newgard C.B., Margulies K.B., Husain M., Inzucchi S.E. (2022). Metabolomic Profiling of the Effects of Dapagliflozin in Heart Failure with Reduced Ejection Fraction: DEFINE-HF. Circulation.

[13] Santos-Gallego C.G., Mayr M., Badimon J. (2022). SGLT2 Inhibitors in Heart Failure: Targeted Metabolomics and Energetic Metabolism. Circulation.

[14] Santos-Gallego C.G., Vargas-Delgado A.P., Requena-Ibanez J.A., Garcia-Ropero A., Mancini D., Pinney S., Macaluso F., Sartori S., Roque M., Sabatel-Perez F. (2021). Randomized Trial of Empagliflozin in Nondiabetic Patients with Heart Failure and Reduced Ejection Fraction. J. Am. Coll. Cardiol..

[15] Packer M., Anker S.D., Butler J., Filippatos G., Pocock S.J., Carson P., Januzzi J., Verma S., Tsutsui H., Brueckmann M. (2020). Cardiovascular and Renal Outcomes with Empagliflozin in Heart Failure. N. Engl. J. Med..

[16] Requena-Ibanez J.A., Santos-Gallego C.G., Rodriguez-Cordero A., Vargas-Delgado A.P., Badimon J.J. (2022). Empagliflozin improves quality of life in nondiabetic HFrEF patients. Sub-analysis of the EMPATROPISM trial. Diabetes Metab. Syndr.-Clin. Res. Rev..

[17] Lang R.M., Badano L.P., Mor-Avi V., Afilalo J., Armstrong A., Ernande L., Flachskampf F.A., Foster E., Goldstein S.A., Kuznetsova T. (2015). Recommendations for Cardiac Chamber Quantification by Echocardiography in Adults: An Update from the American Society of Echocardiography and the European Association of Cardiovascular Imaging. J. Am. Soc. Echocardiogr..

[18] Lancellotti P., Tribouilloy C., Hagendorff A., Popescu B.A., Edvardsens T., Pierard L.A., Badano L., Zamorano J.L., European Assoc Cardiovasc I. (2013). Recommendations for the echocardiographic assessment of native valvular regurgitation: An executive summary from the European Association of Cardiovascular Imaging. Eur. Heart J.-Cardiovasc. Imaging.

[19] Mitchell C., Rahko P.S., Blauwet L.A., Canaday B., Finstuen J.A., Foster M.C., Horton K., Ogunyankin K.O., Palma R.A., Velazquez E.J. (2019). Guidelines for Performing a Comprehensive Transthoracic Echocardiographic Examination in Adults: Recommendations from the American Society of Echocardiography. J. Am. Soc. Echocardiogr..

[20] Volpicelli G., Mussa A., Garofalo G., Cardinale L., Casoli G., Perotto F., Fava C., Frascisco M. (2006). Bedside lung ultrasound in the assessment of alveolar-interstitial syndrome. Am. J. Emerg. Med..

[21] Holland A.E., Spruit M.A., Troosters T., Puhan M.A., Pepin V., Saey D., McCormack M.C., Carlin B.W., Sciurba F.C., Pitta F. (2014). An official European Respiratory Society/American Thoracic Society technical standard: Field walking tests in chronic respiratory disease. Eur. Respir. J..

[22] Kroenke K., Spitzer R.L., Williams J.B.W. (2001). The PHQ-9-Validity of a brief depression severity measure. J. Gen. Intern. Med..

[23] Bhatt K.N., Kalogeropoulos A.P., Dunbar S.B., Butler J., Georgiopoulou V.V. (2016). Depression in heart failure: Can PHQ-9 help?. Int. J. Cardiol..

[24] Flint K.M., Fairclough D.L., Spertus J.A., Bekelman D.B. (2019). Does heart failure-specific health status identify patients with bothersome symptoms, depression, anxiety, and/or poorer spiritual well-being?. Eur. Heart J.-Qual. Care Clin. Outcomes.

[25] Rector T.S., Tschumperlin L.K., Kubo S.H., Bank A.J., Francis G.S., McDonald K.M., Cohn J.N. (1994). Clinically Significant Improvements in the Living with Heart-Failure Questionnaire Score as Judged by Patients with Heart-Failure. Qual. Life Res..

[26] Behlouli H., Feldman D.E., Ducharme A., Frenette M., Giannetti N., Grondin F., Michel C., Sheppard R., Pilote L. Identifying relative Cut-Off Scores with Neural Networks for Interpretation of the Minnesota Living with Heart Failure Questionnaire. Proceedings of the Annual International Conference of the IEEE-Engineering-in-Medicine-and-Biology-Society.

[27] Rutledge T., Reis V.A., Linke S.E., Greenberg B.H., Mills P.J. (2006). Depression in heart failure-A meta-analytic review of prevalence, intervention effects, and associations with clinical outcomes. J. Am. Coll. Cardiol..

[28] Sokoreli I., Vries J.J.G., Pauws S.C., Steyerberg E.W. (2016). Depression and anxiety as predictors of mortality among heart failure patients: Systematic review and meta-analysis. Heart Fail. Rev..

[29] Regan J.A., Kitzman D.W., Leifer E.S., Kraus W.E., Fleg J.L., Forman D.E., Whellan D.J., Wojdyla D., Parikh K., O′onnor C.M. (2019). Impact of Age on Comorbidities and Outcomes in Heart Failure with Reduced Election Fraction. JACC-Heart Fail..

[30] Sunbul E.A., Sunbul M., Yanartas O., Cengiz F., Bozbay M., Sari I., Gulec H. (2016). Increased Neutrophil/Lymphocyte Ratio in Patients with Depression is Correlated with the Severity of Depression and Cardiovascular Risk Factors. Psychiatry Investig..

[31] Lin T.K., Hsu B.C., Li Y.D., Chen C.H., Lin J.W., Chien C.Y., Weng C.Y. (2019). Prognostic Value of Anxiety Between Heart Failure with Reduced Ejection Fraction and Heart Failure with Preserved Ejection Fraction. J. Am. Heart Assoc..

[32] Ceriani E., Casazza G., Peta J., Torzillo D., Furlotti S., Cogliati C. (2020). Residual congestion and long-term prognosis in acutely decompensated heart failure patients. Intern. Emerg. Med..

[33] Cascino T.M., Kittleson M.M., Lala A., Stehlik J., Palardy M., Pamboukian S.V., Ewald G.A., Mountis M.M., Horstmanshof D.A., Robinson S.W. (2020). Comorbid Conditions and Health-Related Quality of Life in Ambulatory Heart Failure Patients REVIVAL (Registry Evaluation of Vital Information for VADs in Ambulatory Life REVIVAL). Circ.-Heart Fail..

[34] Blumenthal J.A., Babyak M.A., O′Connor C., Keteyian S., Landzberg J., Howlett J., Kraus W., Gottlieb S., Blackburn G., Swank A. (2012). Effects of Exercise Training on Depressive Symptoms in Patients With Chronic Heart Failure The HF-ACTION Randomized Trial. JAMA-J. Am. Med. Assoc..

[35] Jeyanantham K., Kotecha D., Thanki D., Dekker R., Lane D.A. (2017). Effects of cognitive behavioural therapy for depression in heart failure patients: A systematic review and meta-analysis. Heart Fail. Rev..

[36] Angermann C.E., Gelbrich G., Stork S., Gunold H., Edelmann F., Wachter R., Schunkert H., Graf T., Kindermann I., Haass M. (2016). Effect of Escitalopramon All-Cause Mortality and Hospitalization in Patients with Heart Failure and Depression The MOOD-HF Randomized Clinical Trial. JAMA-J. Am. Med. Assoc..

